# Physical Activity, Cardiovascular Status, Mortality, and Prediabetes in Hispanic and Non-Hispanic Adults

**DOI:** 10.1001/jamanetworkopen.2024.15094

**Published:** 2024-06-06

**Authors:** Sarah K. Alver, Stephanie Pan, Yasmin Mossavar-Rahmani, Daniela Sotres-Alvarez, Kelly R. Evenson, James S. Floyd, Vanessa Xanthakis, Juan Lin, Carmen Cuthbertson, Linda C. Gallo, Jianwen Cai, Frank J. Penedo, Maria M. Llabre, Kunihiro Matsushita, Gregory A. Talavera, Amber Pirzada, Nicole Spartano, Martha L. Daviglus, Ramachandran S. Vasan, Robert C. Kaplan

**Affiliations:** 1 Public Health Sciences Division, Fred Hutchinson Cancer Center, Seattle, Washington; 2 Department of Biostatistics, Boston University School of Public Health, Boston, Massachusetts; 3 Department of Epidemiology & Population Health, Albert Einstein College of Medicine, Bronx, New York; 4 Department of Biostatistics, Gillings School of Global Public Health, University of North Carolina at Chapel Hill, Chapel Hill; 5 Department of Epidemiology, Gillings School of Global Public Health, University of North Carolina at Chapel Hill, Chapel Hill; 6 Cardiovascular Health Research Unit, Department of Medicine, University of Washington, Seattle; 7 Department of Epidemiology, University of Washington, Seattle; 8 National Heart, Lung, and Blood Institute’s and Boston University’s Framingham Heart Study, Framingham, Massachusetts; 9 Section of Preventive Medicine and Epidemiology, Boston University Chobanian & Avedisian School of Medicine, Boston, Massachusetts; 10 Health Education and Promotion, College of Health and Human Performance, East Carolina University, Greenville, North Carolina; 11 Department of Psychology, San Diego State University, San Diego, California; 12 Department of Medicine, University of Miami, Miami, Florida; 13 Department of Psychology, University of Miami, Miami, Florida; 14 Department of Epidemiology, Johns Hopkins Bloomberg School of Public Health, Johns Hopkins University, Baltimore, Maryland; 15 Division of Cardiology, Johns Hopkins School of Medicine, Johns Hopkins University, Baltimore, Maryland; 16 Institute for Minority Health Research, University of Illinois Chicago, Chicago; 17 Section of Endocrinology, Diabetes, Nutrition, and Weight Management, Boston University Chobanian & Avedisian School of Medicine, Boston, Massachusetts; 18 University of Texas School of Public Health, San Antonio; 19 University of Texas Health Science Center, San Antonio

## Abstract

**Importance:**

Data are limited on the association of physical activity (PA) with incident cardiovascular disease (CVD) and mortality in prediabetes, especially in racial and ethnic minority groups, including Hispanic and Latino populations.

**Objective:**

To determine the association of PA with incident CVD and mortality by prediabetes status among Hispanic or Latino and non-Hispanic adults.

**Design, Setting, and Participants:**

This cohort study included data from 2 cohorts of adults with prediabetes or normoglycemia who were free of CVD at baseline visit: the Hispanic Community Health Study/Study of Latinos (HCHS/SOL) from baseline examination through 2017, with median (IQR) follow-up of 7.8 (7.2-8.5) years, and the Framingham Heart Study (FHS) with non-Hispanic participants from index examination through 2019, with median (IQR) follow-up of 9.6 (8.1-10.7) years. Analyses were conducted between September 1, 2022, and January 10, 2024.

**Exposure:**

The primary exposure was baseline accelerometry-measured moderate to vigorous PA, insufficient vs sufficient to meet 2018 Physical Activity Guidelines for Americans (PAG) in both cohorts; additional accelerometer-measured exposures in HCHS/SOL were steps per day, sedentary behavior, and counts per min.

**Main Outcomes and Measures:**

The outcome was a composite of incident CVD or all-cause mortality, whichever came first.

**Results:**

This cohort study included 13 223 participants: from HCHS/SOL, there were 9456 adults (all self-identified Hispanic or Latino ethnicity; survey-adjusted mean [SD] age, 38.3 [13.9] years, unweighted counts 5673 (60.0%) female; 4882 [51.6%] with normoglycemia; 4574 [48.4%] with prediabetes), and from FHS there were 3767 adults (3623 [96.2%] non-Hispanic and 140 [3.7%] Hispanic or Latino ethnicity, with 4 [0.1%] participants missing ethnicity; mean [SD] age, 54.2 [13.6] years; 2128 (56.5%) female; 2739 [72.7%] with normoglycemia; 1028 [27.3%] with prediabetes). Not meeting PAG was associated with higher risk of the composite outcome among participants with normoglycemia (vs PAG met; hazard ratio [HR], 1.85 [95% CI, 1.12-3.06]), but not among participants with prediabetes (HR, 1.07 [95% CI, 0.72-1.58]). For HCHS/SOL, no statistically significant association was found between the composite outcome and other PA metrics, although estimated HRs tended to be higher for lower activity in the normoglycemia group but not for the prediabetes group (eg, for steps less than vs at least 7000 per day, the HR was 1.58 [95% CI, 0.85-2.93] for normoglycemia vs 1.08 [95% CI 0.67-1.74] for prediabetes). While there was also no association in HCHS/SOL between the composite outcome and sedentary behavior, results were similar in the prediabetes group (HR per 30 minutes per day of sedentary behavior, 1.05 [95% CI 0.99-1.12]) and in the normoglycemia group (HR, 1.07 [95% CI 0.98-1.16]).

**Conclusions and Relevance:**

In this cohort study of US Hispanic or Latino and non-Hispanic adults, lower moderate to vigorous PA levels were associated with CVD or mortality among participants with normoglycemia but not participants with prediabetes. Adults with prediabetes may benefit from reducing sedentary behavior and improving multiple lifestyle factors beyond improving moderate to vigorous PA alone.

## Key Points

**Question:**

Is meeting Physical Activity Guidelines for Americans (PAG) associated with cardiovascular disease (CVD) or mortality risk similarly for individuals with prediabetes and normoglycemia, and do the associations of activity with health outcomes differ between Hispanic or Latino and non-Hispanic persons?

**Findings:**

In this cohort study of 13 223 adults with prediabetes or normoglycemia, not meeting PAG was associated with CVD or mortality risk in adults with normoglycemia but not in individuals with prediabetes in Hispanic or Latino and non-Hispanic cohorts.

**Meaning:**

Because meeting PAG was not associated with lower CVD or mortality risk in adults with prediabetes, individuals may need to improve multiple lifestyle factors, including reducing sedentary behavior.

## Teaser

This cohort study examines whether meeting Physical Activity Guidelines for Americans is associated with cardiovascular disease or mortality risk similarly among individuals with prediabetes and normoglycemia, and whether physical activity associations with health outcomes differ between Hispanic or Latino and non-Hispanic adults.

## Introduction

Results from the US Diabetes Prevention Program show that lifestyle interventions can reduce type 2 diabetes incidence in individuals with risk factors.^1^ While the association between mortality and physical activity (PA) has been studied in multiple cohorts,^2,3^ data are limited for persons with prediabetes and Hispanic or Latino groups. Prediabetes is associated with higher risk of mortality and CVD^4^ and is present in 34% of Americans, with estimated age-adjusted prevalence of 35.3% overall in Hispanic or Latino persons.^5^ Characteristics of Hispanic or Latino persons, such as high workplace exertion, may differ from the overall US population, justifying the need for Hispanic- or Latino-specific studies.^6^ More US data are needed on the effects of lifestyle interventions on health outcomes in prediabetes over longer follow-up, particularly in populations with a high burden of diabetes, which includes US Hispanic or Latino groups.^7^

Our primary goal was to determine the association between PA and the composite outcome of all-cause mortality and incident cardiovascular disease (CVD) by prediabetes status and background: Hispanic or Latino and non-Hispanic. We studied this association in adults from the Hispanic Community Health Study/Study of Latinos (HCHS/SOL) and from the primarily non-Hispanic White adults in the Framingham Heart Study (FHS) using accelerometer-measured moderate to vigorous PA (MVPA), the PA measure most readily harmonized between the 2 cohorts. To further study the association between movement and the composite outcome in Hispanic or Latino persons, we studied accelerometer-measured sedentary behavior, mean number of steps per day, mean accelerometer counts per minute, and calibrated activity-related energy expenditure (CAEE), which accounts for PA measurement error,^8^ in HCHS/SOL.

## Methods

### HCHS/SOL and FHS Study Cohorts

The 2 cohort studies were approved by institutional review boards at each field center where all participants gave written informed consent. The present study conformed to the Strengthening the Reporting of Observational studies in Epidemiology (STROBE) reporting guideline,^9^ and where applicable, the Preferred Reporting Items for Systematic Reviews and Meta-Analysis (PRISMA)^10^ guideline.

The HCHS/SOL and FHS collected in-person baseline examination data and incident events data during follow-up.^11,12^ The HCHS/SOL is a community-based prospective cohort study of 16 415 self-identified Hispanic or Latino persons aged 18 to 74 years at screening from randomly selected households in four US field centers (Chicago, Illinois; Miami, Florida; Bronx, New York; and San Diego, California) during 2008 to 2011.^13^ The HCHS/SOL used a stratified 2-stage probability sample, with the first sampling stage involving randomly selected census block groups, and the second sampling stage involving random selection of household units within the selected geographic areas.^14^

The FHS participants^11^ included the Generation 2 (Gen 2) cohort enrolled in 1971 (the offspring [and their spouses] of the original cohort who were sampled in the 1940s in Framingham, Massachusetts),^15^ Gen 3 and new offspring spouses (NOS) cohorts enrolled in 2002 (children of the Gen 2 and spouses of Gen 2 not already enrolled in FHS),^12^ and the Omni 1 and Omni 2 cohorts, who were recruited in 1994 and 2003, respectively, to reflect the greater ethnic diversity of Framingham.^16^ Our initial sample included 4381 participants from Gen 3, NOS, Omni 2, Gen 2, and Omni 1 who completed the index examination and had accelerometer data.

### Assessment of PA/Sedentary Behavior

The FHS^17^ and HCHS/SOL^18,19^ used similar hip-worn accelerometers (Actical model 198-0200-03 in HCHS/SOL; Actical model 198-0200-00 in FHS; Respironics) to measure at least 7 days’ PA at baseline. In HCHS/SOL, accelerometer counts classified sedentary behavior as fewer than 100 counts/min and MVPA as at least 1535 counts/min^18,19^ with 1 minute epoch length. A subset of HCHS/SOL participants had activity-related energy expenditure measured using indirect calorimetry and doubly labeled water, enabling the derivation of CAEE for HCHS/SOL participants.^8^ The FHS accelerometer data were recorded in 30-second epochs, with MVPA classified as at least 1535 counts/min.^17^ Accelerometer data were preprocessed by removing the time the device was not worn using the Choi et al algorithm.^20^

### Inclusion Criteria

**Figure 1.**
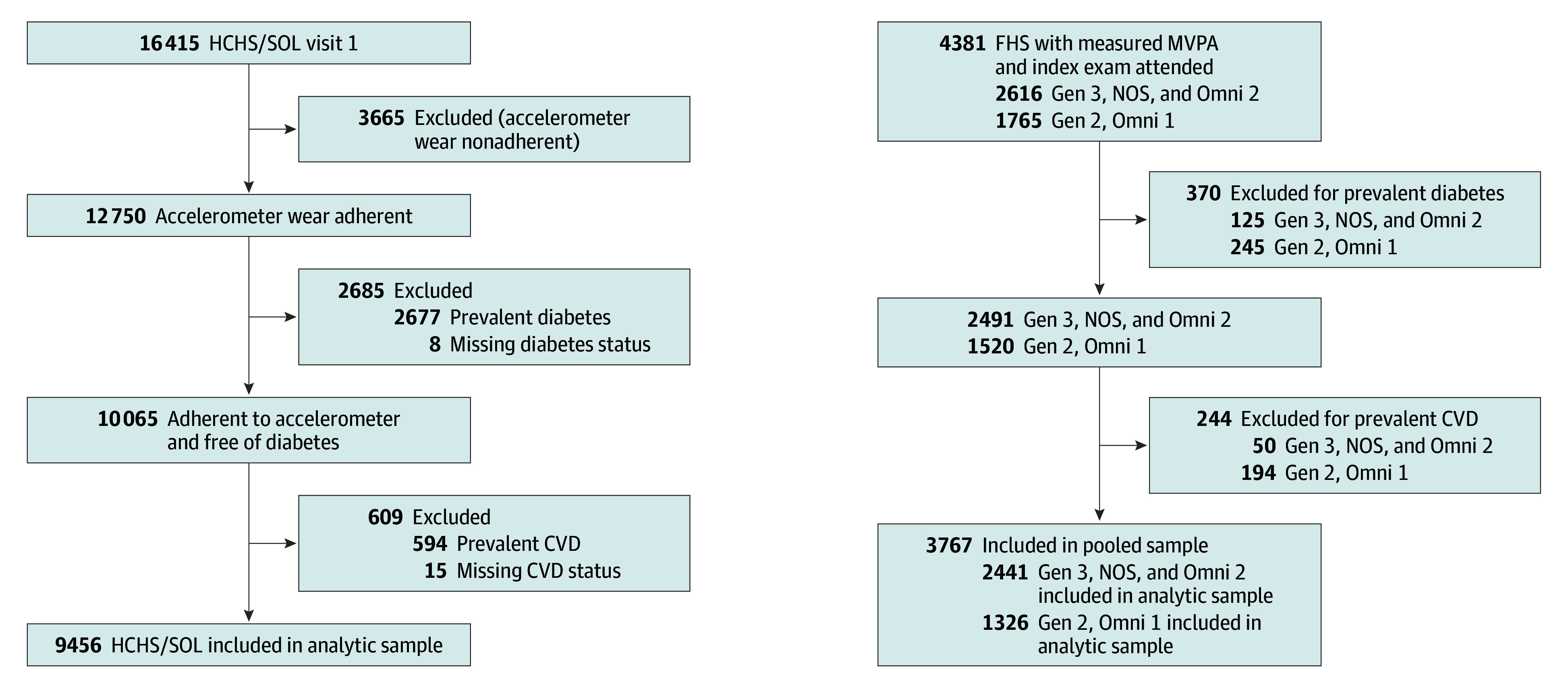
Sample Derivation for Hispanic Community Health Study/Study of Latinos (HCHS/SOL) and Framingham Heart Study (FHS) CVD indicates cardiovascular disease; Gen, generation; MVPA, moderate to vigorous physical activity; NOS, new offspring spouses.

Figure 1 depicts sample derivation for both cohorts. We excluded participants who had prevalent diabetes or CVD at baseline or the index examination, in 2008 through 2011 for HCHS/SOL, and in 2008 through 2011 (examination 2, Gen 3, NOS and Omni 2) or in 2011 through 2014 (examination 9 or examination 4, Gen 2 or Omni 1) for FHS. We excluded participants having fewer than 3 accelerometer days with wear times at least 10 hours per day.

For HCHS/SOL, we used self-reported diagnosis of diabetes plus American Diabetes Association laboratory criteria to classify diabetes and prediabetes at baseline. Laboratory-based criteria were fasting more than 8 hours with glucose levels at least 126 mg/dL (100-125 mg/dL for prediabetes) (to convert serum glucose levels to millimoles per liter, multiply by 0.0555), or fasting 8 hours or less with glucose levels 200 mg/dL or higher, or glucose levels for an oral glucose tolerance test at least 200 mg/dL (140-199 mg/dL for prediabetes), or hemoglobin A_1C_ levels at least 6.5% of total hemoglobin (≥5.7% but <6.5% for prediabetes) (to convert to proportion of total hemoglobin, multiply by 0.01). The FHS used the same laboratory criteria or current diabetes treatment to classify glycemic status at baseline.

For the HCHS/SOL sample, baseline prevalent CVD was defined as electrocardiogram findings of possible old myocardial infarction (MI), self-reported history of MI, congestive heart failure, stroke, transient ischemic attack (TIA), or revascularization procedure. For the FHS sample, baseline prevalent CVD was defined as coronary heart disease (fatal or nonfatal angina, acute coronary syndrome, coronary insufficiency, or MI), cerebrovascular event (stroke or transient ischemic attack), heart failure, and peripheral arterial disease (intermittent claudication).

### Outcomes

The composite outcome was defined as time to incident CVD event or death, whichever came first. Event numbers were insufficient to support separate analyses for each outcome type. Follow-up time was from the baseline examination to proxy-reported death or first adjudicated CVD event through 2017 for HCHS/SOL and from the index examination through 2019 for FHS. In both cohorts, potential events were identified through annual follow-up surveys. When a hospitalization or death was reported, mechanisms to obtain the records were initiated, and physician reviewers classified the events.

In HCHS/SOL, incident CVD was defined as the first adjudicated definite or probable MI, primary diagnosis of stoke or TIA, or definite or probable acute decompensated heart failure requiring hospitalization. For FHS, incident CVD was defined as first new onset MI, congestive heart failure, or stroke or TIA. Incident events for MI included those identified by electrocardiogram or enzyme test results and history or autopsy (silent or not silent). Stroke or TIA included atherothrombotic brain infarction, cerebral hemorrhage, subarachnoid hemorrhage, other cerebrovascular accident, and definite cerebrovascular accident with unknown type.

### Statistical Analysis

#### Association of MVPA With Outcomes

Baseline descriptive statistics were calculated for each glycemic group in FHS and HCHS/SOL. To determine whether higher PA levels were associated with CVD and mortality risk in prediabetes, as with normoglycemia, and to compare this association between Hispanic or Latino and non-Hispanic populations, we fit Cox proportional hazards models for time to first CVD event or death, including a prediabetes by PA interaction term, in each cohort. Since MVPA was readily harmonized between the 2 cohorts and a focus of the Diabetes Prevention Program, it was used as the exposure for this comparison modeled as continuous or binary: sufficient MVPA to meet (≥150 minutes/week; reference group) or not meet (<150 minutes/week; exposed group) the 2018 Physical Activity Guidelines for Americans (PAG).^21^

Minimally adjusted models included age and sex for both cohorts plus field center and Hispanic background for HCHS/SOL. Fully adjusted models added body mass index (BMI, calculated as weight in kilograms divided by height in meters squared), accelerometer wear time, smoking, sleep duration, 12-item Short Form Survey (SF-12) pain score (range 1-5, with higher scores indicating greater interference of pain with a participant's normal work), systolic blood pressure, and low-density lipoprotein cholesterol (LDL-C) levels in FHS participants and the same covariates plus educational level (in years) and work-related PA (self-reported) for HCHS/SOL. All exposures and covariates were measured at baseline only. All models in HCHS/SOL accounted for missing accelerometry data using inverse probability weighting and accounted for the complex survey design. We also performed a random-effects meta-analysis of the results from HCHS/SOL and FHS with subgroup analysis by glycemic classification.

#### Association of Accelerometry Metrics With Outcomes Among HCHS/SOL Participants

To further assess the association of PA and sedentary behavior by glycemic status with the composite outcome in Hispanic or Latino individuals, we fit Cox proportional hazards models with a prediabetes by PA or sedentary behavior interaction term to the HCHS/SOL data using counts per min, sedentary behavior, or steps as the primary exposure, with each modeled as continuous, binary, or in tertiles. Where results appeared consistent across prediabetes and normoglycemia groups, these groups were combined. For binary exposures, steps were classified as fewer than 7000 steps/day vs 7000 steps/day or more, based on findings that 7000 steps/day is approximately consistent with a 150-minute/week MVPA.^22^ The counts per minute were dichotomized about the median value of 148. We dichotomized sedentary behavior as the lowest tertile (≤607 minutes/day) vs the highest 2 tertiles (>607 minutes/day). We explored possible nonlinear associations between the continuous exposures and the composite outcome using restricted cubic splines with 4 knots set at quartiles of each PA and sedentary behavior exposure. Models included prediabetes by PA or prediabetes by SB interaction terms and were adjusted for age, sex, field center, Hispanic or Latino background, systolic blood pressure, LDL-C, BMI, smoking status, alcohol use, educational level, employment status, income, sleep duration, diet quality,^23^ language preference, wear time, physician visit in the last year prior to baseline visit, and use of health insurance. All models were adjusted for complex survey design. As BMI, LDL-C levels, and systolic blood pressure could be considered mediators of the PA outcome association, we fit models in HCHS/SOL for binary and continuous MVPA, steps, counts per minute, and sedentary behavior exposures, with or without these terms, to confirm estimates. We modeled CAEE as a continuous exposure in calibration and outcome models separately for each glycemic group (eMethods in Supplement 1).

#### Sensitivity Analyses and Model Assumptions

Sensitivity analyses for MVPA in FHS and HCHS/SOL excluded participants with BMI lower than 18.5 or events in the first 1 to 2 years of follow-up to check for reverse causation.^24^ We fit separate models for individuals with prediabetes and normoglycemia to compare with interaction models. Proportional hazards assumptions were verified with scaled Schoenfeld residual plots, addressing covariates with violations by stratification.

Statistical analyses were conducted between September 1, 2022, and January 10, 2024, using Stata 17 (StataCorp LLC)^25^ for the meta-analysis and R, version 4.1.3 (R Project for Statistical Computing),^26^ or SAS, version 9.4 (SAS Institute Inc),^27^ for all other analyses. A 2-sided value of *P* < .05 was considered statistically significant.

## Results

### Baseline Characteristics

This cohort study included a total of 13 223 participants; after exclusions (Figure 1), HCHS/SOL had 9456 participants (survey-weighted mean [SD] age, 38.3 [13.9] years; all self-reported Hispanic or Latino ethnicity [unweighted counts], 5673 [60.0%] female and 3783 [40.0%] male, 4882 [51.6%] with normoglycemia; 4574 [48.4%] with prediabetes), and FHS had 3767 participants (mean [SD] age, 54.2 [13.6] years; 3623 [96.2%] non-Hispanic and 140 [3.7%] Hispanic or Latino ethnicity, with 4 [0.1%] missing ethnicity; 2128 [56.5%] female and 1639 [43.5%] male; 2739 [72.7%] with normoglycemia; 1028 [27.3%] with prediabetes). Compared with the normoglycemia groups, the prediabetes groups had fewer females, older age, less alcohol use, fewer individuals in higher education categories, more individuals with hypertension or high cholesterol, and less time in MVPA (Table 1). In HCHS/SOL, individuals with prediabetes vs normoglycemia had shorter sleep duration and higher Alternative Healthy Eating Index 2010 scores (range, 0 [nonadherence] to 110 [perfect adherence]), while these variables were similar between prediabetes and normoglycemia in FHS. In both glycemic groups, BMI was higher in HCHS/SOL than in FHS. Compared with FHS participants, HCHS/SOL participants were younger and more likely to meet PAG. In both cohorts, meeting PAG was associated with favorable socioeconomic and behavioral variables (eTables 1 and 2 in Supplement 1).

**Table 1.** Baseline Characteristics in the Hispanic Community Health Study/Study of Latinos (HCHS/SOL) and Framingham Heart Study (FHS) Pooled Sample, by Glycemic Status

Variable	HCHS/SOL participants, weighted percentages (95% CI)^a^			FHS pooled sample, participants, No. (%)^b^		
	Normoglycemia (n = 4882)	Prediabetes (n = 4574)	*P* value^c^	Normoglycemia (n = 2739)	Prediabetes (n = 1028)	*P* value
Sex						
Female	54.4 (52.3-56.6)	48.9 (46.5-51.2)	.001	1554 (56.7)	574 (55.8)	.62
Male	45.6 (43.4-47.7)	51.1 (48.8-53.5)		1185 (43.3)	454 (44.2)	
Age, y	33.9 (0.3)	44.6 (0.4)	<.001	52.1 (13.3)	59.8 (12.6)	<.001
BMI	27.9 (0.2)	30.3 (0.2)	<.001	26.9 (4.8)	28.9 (5.3)	<.001
Employment status^d^						
Retired	2.1 (1.7-2.6)	8.8 (7.6-10.1)	<.001	350 (13.1)	232 (24.3)	<.001
Not retired/not employed	43.1 (40.8-45.4)	38 (35.7-40.3)		281 (10.5)	93 (9.8)	
Employed part-time	19.6 (17.9-21.2)	16.4 (14.7-18.2)		444 (16.7)	144 (15.1)	
Employed full-time	35.2 (33.2-37.2)	36.7 (34.4-39)		1592 (59.7)	484 (50.8)	
Annual household income,$^e^						
HCHS/SOL						
<10 000	11.5 (9.9-13.2)	12.6 (11.2-14)	.51	NA	NA	NA
10 001-20 000	27.9 (25.8-30)	28.2 (26.1-30.4)		NA	NA	
20 001-40 000	30.6 (28.2-32.9)	31.4 (29.2-33.6)		NA	NA	
40 001-75 000	13.8 (12.1-15.4)	13.8 (11.8-15.8)		NA	NA	
>75 000	6.3 (4.3-8.3)	4.9 (3.6-6.1)		NA	NA	
Not reported	9.9 (8.5-11.3)	9.1 (7.7-10.6)		NA	NA	
FHS						
<20 000	NA	NA	NA	61 (2.2)	27 (2.6)	<.001
20 000-34 999	NA	NA		110 (4)	26 (2.5)	
35 000-54 999	NA	NA		233 (8.5)	67 (6.5)	
55 000-74 999	NA	NA		285 (10.4)	79 (7.7)	
75 000-100 000	NA	NA		382 (14)	110 (10.7)	
>100 000	NA	NA		782 (28.6)	189 (18.4)	
Prefer not to answer or unknown	NA	NA		886 (32.4)	530 (51.6)	
Sleep duration, h	8.1 (0.03)	7.8 (0.03)	<.001	7.2 (1.0)	7.2 (1.2)	.12
Alcohol current (vs former or never)	56.2 (53.5-59)	52.3 (49.8-54.8)	.03	2232 (85.6)	705 (75.2)	<.001
Smoking current (vs former or never)	20.2 (18.4-22)	21.7 (19.7-23.8)	.26	173 (6.3)	87 (8.5)	.02
Educational level^f^						
HCHS/SOL						
<High school	11.6 (10.3-12.8)	21.1 (19.3-22.8)	<.001	NA	NA	NA
High school	44.4 (42-46.8)	40.7 (38.3-43.1)		NA	NA	
Trade school	10.5 (9.2-11.9)	12.1 (10.6-13.5)		NA	NA	
University or other	33.5 (30.8-36.2)	26.1 (24-28.3)		NA	NA	
FHS						
<High school	NA	NA	NA	14 (0.7)	14 (2.5)	<.001
High school/GED	NA	NA		223 (11.3)	75 (13.1)	
Some college, technical, associate	NA	NA		533 (27)	187 (32.8)	
Bachelor's or graduate degree or professional	NA	NA		1204 (61)	295 (51.7)	
Years of education^f^	12.5 (0.1)	11.5 (0.1)	<.001	14.8 (2.6)	14.6 (2.6)	.10
Language preference (English)	30.8 (28.1-33.5)	20.4 (18.2-22.7)	<.001	NA	NA	NA
Depression symptoms						
HCHS/SOL						
CESD-10 score	6.7 (0.1)	6.5 (0.1)	.28	NA	NA	NA
FHS						
CESD-20 score^d^	NA	NA	NA	3.0 (1.0-6.0)	3.0 (1.0-6.5)	.99
AHEI-2010 score^g^	45.9 (0.2)	48.2 (0.2)	<.001	63.3 (13.1)	63.1 (12.7)	.73
Have health insurance, yes (vs no)	47.1 (44.1-50.1)	48.2 (45.5-50.9)	.53	2656 (99.4)	947 (99.1)	.34
Physician visit in last year, yes (vs no)	62.4 (60-64.7)	65.1 (62.5-67.7)	.09	2509 (93.8)	916 (95.5)	.05
Activity or work limited by health^h^	4.9 (3.9-5.8)	6.9 (5.9-8)	.004	NA	NA	NA
SF-12 General Health score^d^	2.7 (0.03)	2.9 (0.02)	<.001	3 (0.7)	2.9 (0.8)	<.001
SF-12 Pain score^d^	1.6 (0.03)	1.7 (0.03)	.04	0.5 (0.8)	0.6 (0.9)	<.001
Hypertension^i^	8.2 (7.2-9.1)	23.7 (21.7-25.6)	<.001	718 (26.2)	455 (44.3)	<.001
Systolic blood pressure, mm Hg	114.5 (0.3)	122.7 (0.4)	<.001	117.9 (15.2)	123.3 (15.7)	<.001
Hypercholesterolemia^j^	28.2 (26.1-30.3)	48.5 (46.2-50.8)	<.001	794 (29)	494 (48.1)	<.001
LDL-C, mg/dL	113.9 (0.8)	128 (0.9)	<.001	104.1 (29.2)	109.1 (30.9)	<.001
MVPA, min/d	27.7 (0.8)	24 (0.9)	<.001	15.8 (6.8-29.0)	10.2 (3.4-21.5)	<.001
Met 2018 MVPA Guidelines	48.9 (46.2-51.5)	40.5 (38-43)	<.001	828 (36.0)	234 (25.6)	<.001
Work-related PA (self-reported), min/d	83.9 (4.1)	84.4 (3.6)	.92	NA	NA	NA

Abbreviations: AHEI-2010, Alternative Healthy Eating Index 2010; CESD-10 and CESD-20, 10- or 20-item Center of Epidemiologic Studies Depression Scale; GED, General Educational Development; LDL-C, low-density lipoprotein cholesterol; MVPA, moderate to vigorous physical activity; NA, not applicable; NOS, new offspring spouses; PA, physical activity; SF-12, 12-item Short Form Survey.

SI conversion factors: To convert total cholesterol, LDL-C, or high-density lipoprotein cholesterol levels to millimoles per liter, multiply by 0.0259.

^a^ Continuous variables are reported as survey-adjusted mean (SE) or median (IQR). Categorical variables are reported as weighted percentages (95% CI).

^b^ Pooled sample from FHS comprises generation (Gen) 2, Gen 3, Omni 1, Omni 2, and NOS; continuous variables are reported as mean (SD) or median (Q1, Q3). Categorical variables are reported as No. (%).

^c^ Values of *P* from survey adjusted *T *tests for continuous variables and χ^2^ tests for categorical variables.

^d^ Employment status, SF-12 General Health score (range 1-5, with higher scores indicating self-report of poorer health), SF-12 Pain score (range 1-5, with higher scores indicating more interference of pain with normal work), CESD-10 score (range 0-30, with higher values indicating more frequent depressive symptoms), CESD-20 score (range, range 0-60, with higher values indicating more frequent depressive symptoms) not available for FHS Omni 1 examination 4.

^e^ Household income is reported for Gen 3/NOS/Omni 2.

^f^ In FHS, education level reported for Gen 3, NOS, Omni 1 and Omni 2; years of education only reported for Gen 2.

^g^ AHEI-2020 scores, ranging from 0 (nonadherence) to 110 (perfect adherence), was not available for NOS/Omni 2.

^h^ SF-12 component “limited in work or other activities due to physical health” response of “most or all of the time.”

^i^ Hypertension defined as systolic blood pressure 140 mm Hg or higher, diastolic blood pressure 90 mm Hg or higher, or taking antihypertensive medication in the last 4 weeks.

^j^ Hypercholesterolemia defined as total cholesterol 240 mg/dL or higher or LDL 160 mg/dL or higher or HDL less than 40 mg/dL or use of lipid lowering drugs.

### MVPA Association With Composite Outcome in HCHS/SOL and FHS

**Figure 2.**
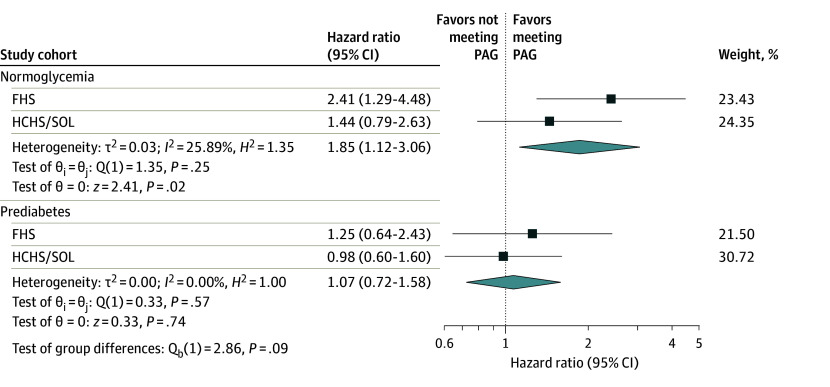
Hazard Ratios for the Composite Outcome of All-Cause Mortality and Incident Cardiovascular Disease With Sufficient (Reference) vs Insufficient (Exposure) Moderate to Vigorous Physical Activity to Meet 2018 Physical Activity Guidelines, by Glycemic Status in the Framingham Heart Study (FHS) and Hispanic Community Health Study/Study of Latinos (HCHS/SOL) The meta-analysis combined glycemic groups across both studies using the random-effects DerSimonian and Laird model. The overall test of group differences shown is for prediabetes vs normoglycemia (see Methods for details). The estimates of between-study variance (τ^2^) are imprecise with only 2 studies. PAG indicates Physical Activity Guidelines for Americans.

Median (IQR) follow-up time in HCHS/SOL was 7.8 (7.2-8.5) years, with 252 events (2.7%), and in FHS, it was 9.6 (8.1-10.7) years, with 195 events (5.2%) (eTable 3 in Supplement 1). Figure 2 depicts estimated hazard ratios (HRs) and 95% CIs for not meeting vs meeting PAG by prediabetes status in FHS and HCHS/SOL. Normoglycemia groups had increased risk of incident CVD or mortality with PAG not met vs met, with HRs of 2.41 (95% CI, 1.29-4.48) for FHS and 1.44 (0.79-2.63) for HCHS/SOL. The prediabetes groups had estimated HRs for PAG not met vs met of 1.25 (95% CI, 0.64-2.43) in FHS and 0.98 (95% CI, 0.60-1.60) in HCHS/SOL. The interactions between prediabetes and PA were not statistically significant. Minimally adjusted and fully adjusted model results were similar (eTable 4 in Supplement 1).

In the meta-analysis, HCHS/SOL included 221 events among 8600 participants (141 events among 4178 participants for prediabetes; 80 events among 4422 participants for normoglycemia). The meta-analysis for FHS included 156 events among 2858 participants (74 events among 762 participants for prediabetes; 92 events among 2096 participants for normoglycemia). The random-effects meta-analysis produced an overall HR for not meeting vs meeting PAG of 1.85 (95% CI, 1.12-3.06) in the normoglycemia group and 1.07 (95% CI, 0.72-1.58) in the prediabetes group (Figure 2). The test statistic for difference in HR between prediabetes and normoglycemia was 2.86 (χ^2^ distribution, 1 *df*; *P* = .09). Heterogeneity between cohorts within the prediabetes and normoglycemia subgroups was not statistically significant.

### Association of Outcomes With Other Accelerometer Measures in HCHS/SOL

Baseline characteristics of HCHS/SOL participants with lower vs higher counts per minute or steps largely mirrored the differences reported for not meeting vs meeting PAG (eTables 6 and 7 in Supplement 1). However, high sedentary behavior had additional correlates in HCHS/SOL beyond those associated with low PA: highest education level, English as preferred language, had health insurance, and had a physician visit within the last year (eTable 5 in Supplement 1). No statistically significant associations were found between the composite outcome and the additional measures in HCHS/SOL, although estimated HRs for the putative “unhealthy” vs “healthy” activity levels were consistently farther from the null in the normoglycemia group, favoring the “healthier” behavior (eg, HR, 1.23 [95% CI 0.69-2.21] in the normoglycemia group and 1.03 [95% CI 0.63-1.69] in the prediabetes group, for counts per minute below vs above the median) (Table 2).

**Table 2.** Association of Composite Outcome of All-Cause Mortality or First Incident Cardiovascular Disease Event With Binary Levels of PA or Sedentary Behavior by Glycemic Status in the Hispanic Community Health Study/Study of Latinos^a^

PA or sedentary behavior metric	Normoglycemia, HR (95% CI)		Prediabetes, HR (95% CI)		*P* value for interaction
	Reference group	Exposed group	Reference group	Exposed group	
MVPA^b^	PAG met	PAG not met	PAG met	PAG not met	
	1	1.57 (0.85-2.88)	1	1.03 (0.64-1.65)	.28
CPM	Above median	Below median	Above median	Below median	
	1	1.23 (0.69-2.21)	1	1.03 (0.63-1.69)	.66
Steps	≥7000/d	<7000/d	≥7000/d	<7000/d	
	1	1.58 (0.85-2.93)	1	1.08 (0.67-1.74)	.32
Sedentary behavior	1st tertile (less sedentary)	2nd or 3rd tertile	1st tertile (less sedentary)	2^nd^ or 3rd tertile	
	1	1.94 (0.84-4.47)	1	1.10 (0.60-2.03)	.19

Abbreviations: CPM, counts per min; HR, hazard ratio; MVPA, moderate to vigorous physical activity; PA, physical activity; PAG, 2018 Physical Activity Guidelines for Americans.

^a^ Data are from 9456 participants in all analyses except for steps, which was among 9421 participants.

^b^ MVPA model shown here had more covariates and more granular categories than the model used for comparison with the Framingham Heart Study shown in Figure 2. Models included a prediabetes by PA or sedentary behavior interaction term and were adjusted for age, sex, field center, Hispanic or Latino background, systolic blood pressure, low-density lipoprotein cholesterol, body mass index, smoking status, alcohol use, educational level, employment status, income, sleep duration, diet, language preference, accelerometer wear time, recent physician visit, and use of health insurance. All models were adjusted for complex survey design. Interaction terms were not statistically significant although effect sizes appeared different between the 2 glycemic categories.

**Figure 3.**
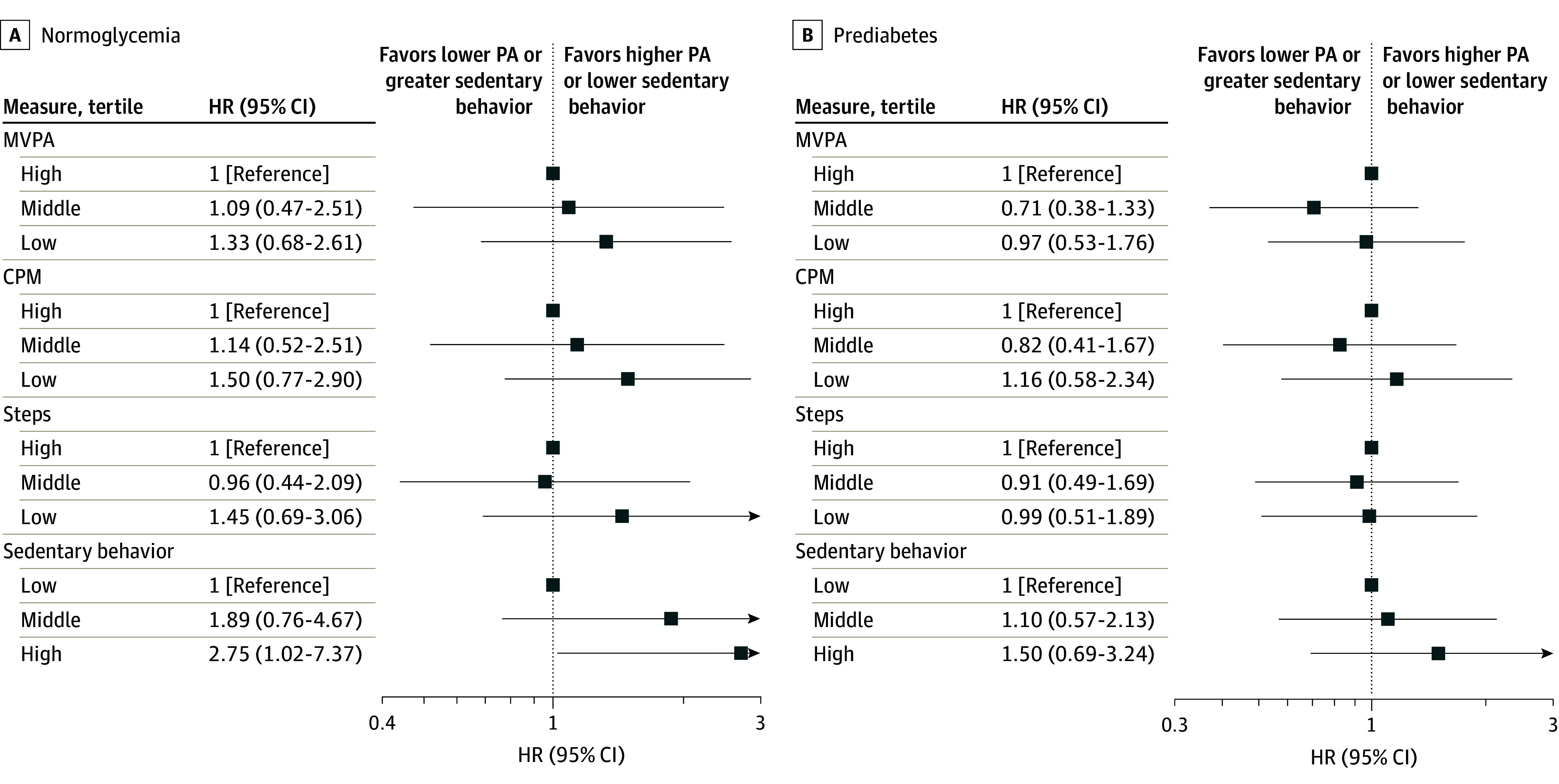
Association of the Composite Outcome of All-Cause Mortality or First Incident Cardiovascular Disease Event With Each Tertile of Physical Activity (PA) or Sedentary Behavior Exposure by Glycemic Status in the Hispanic Community Health Study/Study of Latinos Data from 9456 participants were analyzed except for steps (n = 9421). All models were adjusted for complex survey design. Reference levels were set to be the highest PA or lowest sedentary behavior level. Cutoffs for moderate to vigorous PA (MVPA) were mean minutes per day of 9.33 or lower for low, more than 9.33 to 26.00 for middle, and more than 26.00 for high. Cutoffs for mean counts per minute (CPM) were 116 or lower for low, 116 to 189 for middle, and higher than 189 for high. Cutoffs for mean steps per day were 5090 or fewer for low, 5090 to 8460 for middle, and more than 8460 for high. Cutoffs for sedentary behavior were mean minutes per day of 607 or less, more than 607 to 780 for middle, and more than 780 for high. HR indicates hazard ratio.

In the HCHS/SOL normoglycemia group, for 3-category exposure models, we noted a monotonically increasing HR associated with CVD or mortality across declining MVPA and counts-per-minute tertiles (Figure 3). We observed increasing HRs across successively higher SB tertiles in both HCHS/SOL groups (eg, HR, 1.89 [95% CI, 0.76-4.67] for middle sedentary behavior and HR, 2.75 [95% CI, 1.02-7.37] for high sedentary behavior in the normoglycemia groufp). A U-shaped pattern was noted in the prediabetes group between the composite outcome and MVPA (HR, 0.71 [95% CI, 0.38-1.33] for middle MVPA and 0.97 [95% CI, 0.53-1.76] for low MVPA) and counts per minute (HR, 0.82 [95% CI, 0.41-1.67] for middle counts per minute and HR, 1.16 [95% CI, 0.58-2.34] for low counts per minute). Interaction terms were not statistically significant.

For continuous PA or sedentary behavior exposures in HCHS/SOL, HRs were near 1.00 (eTable 8A in Supplement 1). There was no statistically significant nonlinearity in the adjusted restricted cubic spline models. For each 50 kcal/day CAEE, the HRs were 0.98 (95% CI 0.85-1.15) in the prediabetes group and 0.98 (0.86-1.12) in the normoglycemia group (eTable 8A in Supplement 1). Because HRs for sedentary behavior, steps, and CAEE were of similar magnitude and direction for both glycemic groups, we fit additional fully adjusted models combining glycemic groups. For sedentary behavior, the HR for the composite outcome in the combined group was 1.06 (95% CI, 0.99-1.13) per 30 minutes of sedentary behavior; *P* = .10 (eTable 8B in Supplement 1).

### Results From Sensitivity Analyses

With individuals with low BMI excluded, the results were similar to those in Figure 2. With participants excluded who had events within the first 1 or 2 years, PA-related HRs were similar but of smaller magnitude in both HCHS/SOL glycemic groups (eTable 9 in Supplement 1). For FHS, HRs were smaller in the prediabetes group when excluding individuals with events in the first 1 or 2 years (eTable 10 in Supplement 1). Separate models for the 2 glycemic groups gave results similar to those from the interaction models.

## Discussion

In this cohort study of Hispanic or Latino adults from HCHS/SOL and largely non-Hispanic White adults from FHS, participants with normoglycemia who did not achieve at least 150 minutes/week of MVPA had a greater risk of mortality and CVD than those who met this target. Yet this association was not observed in adults with prediabetes. This pattern of results was observed in each cohort separately and in a meta-analysis of both cohorts. In HCHS/SOL, the association of the composite outcome with other PA measures was largely nonsignificant. The HRs for lower counts per minute and for steps were higher in the normoglycemia group than in the prediabetes group (Table 2), whereas the HR for greater sedentary behavior was in the same direction for the 2 glycemic groups (Figure 3 and eTable 8 in Supplement 1).

Results from several randomized clinical trials suggested that lifestyle modifications, including PA, may not reduce mortality and CVD risk in people with prediabetes.^4,28,29^ The Finnish Diabetes Prevention Study,^30^ Da Qing Diabetes Prevention Study,^31,32^ US Diabetes Prevention Program^1,33,34^ and the Indian Diabetes Prevention Programme^35^ all found that interventions of diet or exercise compared with control (routine or limited advice) reduced risk of progression from prediabetes to diabetes and in some cases improved CVD risk factors. However, none of these interventions had a clear effect on mortality or CVD events over follow-up times from 2.5 to 21 years.

A meta-analysis^36^ combining these 4 randomized controlled trials^1,30,31,32,33,34,35^ with 6 randomized clinical trials^37,38,39,40,41,42,43^ testing pharmacological interventions in persons with prediabetes reported that despite their success in slowing progression to diabetes, the interventions were not successful in reducing all-cause or cardiovascular mortality, except possibly stroke.^36^ The intervention and follow-up periods may have been too brief to influence mortality.^36^ When follow-up of the Da Qing study was extended to 23 years^44^ and to 30 years,^45^ with mean (SD) ages reaching 71.8 (6.9) years (control group) and 70.5 (6.6) years (intervention group), the trial found reduced CVD risk and CVD-related mortality in their lifestyle intervention group compared with the control group (routine advice).

What may explain our observation that decreased PA was associated with mortality and CVD risk in normoglycemia but not significantly so in prediabetes? First, common comorbidities with prediabetes may be relatively resistant to lifestyle changes. In HCHS/SOL, we previously observed favorable 6-year patterns in LDL-C level with lower sedentary behavior, but only in the absence of diabetes, prediabetes, and CVD.^46^ Greater use of medications (antihypertensives, lipid-lowering agents) in prediabetes than in normoglycemia may overshadow the influence of lifestyle on CVD or mortality risk.

Second, chronic inflammation and undiagnosed conditions may be more common in individuals with prediabetes than with normoglycemia,^4^ possibly limiting their activity or increasing risk of adverse events from intense exercise. The threshold of exercise intensity needed to improve cardiorespiratory fitness may vary depending on fitness level, so intensity is difficult to define from accelerometry.^47^ Thus, total PA volume may be more important than intensity in persons with prediabetes. This could partially explain our finding of higher (albeit nonsignificant) HRs for the association of increased sedentary behavior with CVD and mortality risk in both glycemic groups, since sedentary behavior reflects the complement of total time in any measured PA intensity.

Third, activity level could be differentially misclassified between prediabetes and normoglycemia groups. Activity was measured at baseline, and participants may have changed activity levels during the follow-up period,^48^ which could bias findings despite adjustment for multiple covariates.

The studies supporting earlier versions of PAG were based on self-reported PA, although 2018 guidelines also cited studies that employed accelerometers to study PA.^49^ The proportion of people who meet PAG targets measured in minutes per week is lower when defined using accelerometer data vs self-reported PA.^50^ Thus, some participants’ PA levels may be misclassified. Nevertheless, we found similar associations between MVPA and the outcome with MVPA modeled continuously or in tertiles.^49,50^

### Limitations and Strengths

Study limitations included our inability to capture progression to diabetes as an intermediary end point due to lack of intercurrent diabetes assessments prior to events. In a study of 45 782 Asian individuals with prediabetes, at any given level of PA, progression to diabetes was a leading predictor of mortality.^51^ As with the Da Qing study, more benefit in the prediabetes group may occur after longer follow-up.^32,44,45^ Additionally, while we carefully harmonized data from FHS and HCHS/SOL, unmeasured differences may exist. Finally, several prediabetes by PA interactions were not statistically significant but may have reached significance with more events. The strengths of our study include use of accelerometry-measured PA and replication of findings for MVPA between a Hispanic or Latino cohort and a largely non-Hispanic White cohort.

## Conclusions

The results of this cohort study underscore the importance of preventing prediabetes, which itself is associated with higher risk of mortality and CVD. The apparent greater benefit of PA in those with normoglycemia than in those with prediabetes suggests that solely increasing PA may not overcome the increased risk of mortality and CVD associated with prediabetes. Emphasis on decreasing sedentary behavior and improving multiple lifestyle factors may benefit adults with prediabetes.

## Notes

Supplement 1. **eMethods.** Details of methods for calibrated activity-related energy expenditure models **eTable 1.** Baseline characteristics by 2018 PAG Met/Not Met, HCHS/SOL **eTable 2.** Baseline characteristics by 2018 PAG Met/Not Met, FHS **eTable 3.** Number of incident events stratified by prediabetes status, FHS and HCHS/SOL **eTable 4.** Estimated hazard ratio (95% confidence interval) for the association of the combined outcome of all-cause mortality or incident CVD event with binary PA/sedentary behavior exposure by glycemic status, FHS and HCHS/SOL, minimally adjusted models **eTable 5.** Baseline characteristics at low and high sedentary behavior levels, HCHS/SOL **eTable 6.** Baseline characteristics at low and high counts per minute, HCHS/SOL **eTable 7.** Baseline characteristics at steps ≥7000/d vs <7000/d, HCHS/SOL **eTable 8.** (A) Estimated hazard ratios (95% confidence intervals) for the association of the combined outcome (all-cause mortality or incident CVD event) with continuous PA or sedentary behavior exposures by glycemic status, HCHS/SOL; (B) Analogous HR for sedentary behavior, steps and CAEE for combined glycemic groups **eTable 9.** Estimated hazard ratios (95% confidence intervals), with additional exclusions as a sensitivity analysis, for the association of the composite outcome of all-cause mortality or first incident CVD event with PA levels (PAG not met as exposure vs met as reference) by glycemic status in HCHS/SOL **eTable 10.** Estimated hazard ratios (95% confidence intervals), with additional exclusions as a sensitivity analysis, for the association of the composite outcome of all-cause mortality or first incident CVD event with PA levels (PAG not met as exposure vs met as reference) by glycemic status in FHS

Supplement 2.
Data Sharing Statement
