# Supplementary material for: Physical Activity, Cardiovascular Status, Mortality, and Prediabetes in Hispanic and Non-Hispanic Adults
Source: JAMA Netw Open. 2024 Jun 6;7(6):e2415094. doi: 10.1001/jamanetworkopen.2024.15094 (PMC11157354; doi:10.1001/jamanetworkopen.2024.15094)
Supplement: Supplement 1. — eMethods. Details of methods for calibrated activity-related energy expenditure models eTable 1. Baseline characteristics by 2018 PAG Met/Not Met, HCHS/SOL eTable 2. Baseline characteristics by 2018 PAG Met/Not Met, FHS eTable 3. Number of incident events stratified by prediabetes status, FHS and HCHS/SOL eTable 4. Estimated hazard ratio (95% confidence interval) for the association of the combined outcome of all-cause mortality or incident CVD event with binary PA/sedentary behavior exposure by glycemic status, FHS and HCHS/SOL, minimally adjusted models eTable 5. Baseline characteristics at low and high sedentary behavior levels, HCHS/SOL eTable 6. Baseline characteristics at low and high counts per minute, HCHS/SOL eTable 7. Baseline characteristics at steps ≥7000/d vs <7000/d, HCHS/SOL eTable 8. (A) Estimated hazard ratios (95% confidence intervals) for the association of the combined outcome (all-cause mortality or incident CVD event) with continuous PA or sedentary behavior exposures by glycemic status, HCHS/SOL; (B) Analogous HR for sedentary behavior, steps and CAEE for combined glycemic groups eTable 9. Estimated hazard ratios (95% confidence intervals), with additional exclusions as a sensitivity analysis, for the association of the composite outcome of all-cause mortality or first incident CVD event with PA levels (PAG not met as exposure vs met as reference) by glycemic status in HCHS/SOL eTable 10. Estimated hazard ratios (95% confidence intervals), with additional exclusions as a sensitivity analysis, for the association of the composite outcome of all-cause mortality or first incident CVD event with PA levels (PAG not met as exposure vs met as reference) by glycemic status in FHS [file jamanetwopen-e2415094-s001.pdf]

Supplementary Online Content

Alver SK, Pan S, Mossavar-Rahmani Y, et al. Physical activity, cardiovascular status, mortality, and prediabetes among Hispanic or Latino and Non-Hispanic adults. *JAMA Netw Open*. 2024;7(6):e2415094. doi:10.1001/jamanetworkopen.2024.15094

eMethods. Details of methods for calibrated activity-related energy expenditure models

eTable 1. Baseline characteristics by 2018 PAG Met/Not Met, HCHS/SOL

eTable 2. Baseline characteristics by 2018 PAG Met/Not Met, FHS

eTable 3. Number of incident events stratified by prediabetes status, FHS and HCHS/SOL

eTable 4. Estimated hazard ratio (95% confidence interval) for the association of the combined outcome of all-cause mortality or incident CVD event with binary PA/SB exposure by glycemic status, FHS and HCHS/SOL, minimally adjusted models

eTable 5. Baseline characteristics at low and high sedentary behavior levels, HCHS/SOL

eTable 6. Baseline characteristics at low and high counts per minute, HCHS/SOL

eTable 7. Baseline characteristics at steps ≥7000/d vs <7000/d, HCHS/SOL

eTable 8. (A) Estimated hazard ratios (95% confidence intervals) for the association of the combined outcome (all-cause mortality or incident CVD event) with continuous PA or SB exposures by glycemic status, HCHS/SOL; (B) Analogous HR for SB, steps and CAEE for combined glycemic groups

eTable 9. Estimated hazard ratios (95% confidence intervals), with additional exclusions as a sensitivity analysis, for the association of the composite outcome of all-cause mortality or first incident CVD event with PA levels (PAG not met as exposure vs met as reference) by glycemic status in HCHS/SOL

eTable 10. Estimated hazard ratios (95% confidence intervals), with additional exclusions as a sensitivity analysis, for the association of the composite outcome of all-cause mortality or first incident CVD event with PA levels (PAG not met as exposure vs met as reference) by glycemic status in FHS

This supplementary material has been provided by the authors to give readers additional information about their work.

© 2024 Alver SK et al. *JAMA Network Open*.

eMethods. Details of methods for calibrated activity-related energy expenditure (CAEE) models

For CAEE, a calibration model for activity-related energy expenditure (AEE) was fit on a subset of Hispanic Community Health Study/Study of Latinos (HCHS/SOL) participants who were in the Study of Latinos: Nutrition & Physical Activity Assessment Study (SOLNAS). These participants have measured activity-related energy expenditure available – calculated from indirect calorimetry and doubly-labelled water, and the calibration model allows this measure (AEE) to be estimated, i.e. calibrated from available accelerometry measured physical activity (PA) and other covariates, for the rest of the study participants (see Shaw et al 2019 [1] for additional details). For better interpretability, AEE was scaled either by 50 kcal/d, to represent a small increment of PA, or by its standard deviation, specific to each glycemic group (312.2 in the SOLNAS prediabetes group or 307.7 in the SOLNAS normoglycemia group). Then, CAEE was used a measurement-error corrected exposure in Cox proportional hazard models (i.e. the outcome models) for the composite outcome similarly to the other accelerometry-measured exposure variables in this study. We modeled the association of CAEE with the composite outcome using the same covariates as in the calibration model. To keep covariates the same in the calibration and outcome models, CAEE was only modeled as a continuous exposure since it is predicted continuously from the calibration model. Similarly, we fit sets of calibration and outcome models separately for prediabetes and normoglycemia groups to model CAEE rather than fitting an interaction term.

To account for the additional uncertainty introduced by the calibration, the estimated variance was obtained using the resampling based multiple imputation method discussed in Baldoni et al 2021 [2], and then used to calculate confidence intervals for coefficients from the outcome model. We resampled the SOLNAS subset with replacement 500 times and fit the calibration model on each bootstrap set, obtaining 500 sets of regression coefficient for the calibration model. We then used each of those sets of calibration model coefficients to predict CAEE in the rest of the sample and fit the outcome model. The mean of the resulting beta coefficients was used as the point estimate, and the variance of those 500 estimated coefficients plus the mean of their variances in the set of 500 outcome models, was used as the variance. Because AEE is calculated from the calibration model as a continuous variable, and variables should be the same in the calibration and outcome models, we did not model CAEE categorically or involved with an interaction term. Independent variables included in the calibration model were largely those found by Shaw et al [1] to be associated with AEE, and included percent of wear time spent performing light, moderate, and vigorous intensity PA (these three variables treated as the error-prone exposures in the calibration model), and the additional covariates of self-reported sedentary time and work/transportation related PA, age, gender, body mass index (BMI), systolic blood pressure, low density lipoprotein cholesterol, ten-item Center for Epidemiologic Studies Depression Scale, aggregate physical health score, employment status, income with level for missing, smoking status (never, former, current), alcohol use level (none, low, high), education, wear time, Alternative Healthy Eating Index 2010 score, sleep duration, doctor visit in the last year, and use of health insurance. The same covariates were used in the outcome models for CAEE. While generally, height and weight are associated with

© 2024 Alver SK et al. *JAMA Network Open*.

energy expenditure while BMI may be more related to health outcomes, we elected to use BMI in both models. In the SOLNAS subset, there were no participants who had more than one/other Hispanic/Latino background, so a set of calibration and outcome models were fit that excluded those participants from the main study, and another set that collapsed Hispanic/Latino background into 3 categories: Mexican, Caribbean (Cuba, Dominican Republic, and Puerto Rico) and Other. Results were similar regardless of how background was categorized.

© 2024 Alver SK et al. *JAMA Network Open*.

eTable 1. **Baseline characteristics by 2018 PAG Met/Not Met, HCHS/SOL.** HCHS/SOL participant baseline characteristics by Physical Activity Level (2018 PA Guidelines Met/Not Met), n=9456. Mean (SE) or percentage, adjusted for survey design.

	2018 PAG Met (N=4001)	2018 PAG Not Met (N=5455)
**Female sex**	40.2%	62.2%
**Age, yrs**	35.6 (0.3)	40.5 (0.3)
**BMI, kg/m^2**	28.1 (0.1)	29.5 (0.2)
**Employment Status**<br>**Retired**	3.3%	6.2%
**Not retired/not employed**	37.9%	43.6%
**Employed part-time**	20.1%	16.8%
**Employed full-time**	38.6%	33.5%
**Annual Household Income**<br>**Less than $10,000**	11.3%	12.5%
**$10,001-$20,000**	28.1%	28%
**$20,001-$40,000**	31%	30.8%
**$40,001-$75,000**	14.1%	13.5%
**More than $75,000**	6%	5.5%
**Not reported**	9.5%	9.7%
**Sleep Duration**	7.9 (0.03)	8 (0.03)
**Current Alcohol Use**	58.8%	51.2%
**Current Cigarette Use**	21.1%	20.6%
**Education level**<br>**High school**	14.3%	16.4%
**<High school**	46.5%	40%
**Trade school**	8.2%	13.6%
**University/Other**	31%	30%
**Years of education**	12 (0.1)	12.1 (0.1)
**Language Preference (English)**	32.5%	21.6%
**Depression Symptoms (CESD-10)**	6.5 (0.1)	6.7 (0.1)
**AHEI-2010**	47.1 (0.3)	46.7 (0.2)
**Have health insurance**	50.9%	44.8%
**Doctor visit in last yr**	62.4%	64.4%
**Activity/work limited by health**	4.5%	6.7%
**SF-12 General Health Score**	2.7 (0.03)	2.9 (0.02)
**SF-12 Pain Score**	1.6 (0.02)	1.7 (0.03)
**Hypertension**	11.3%	17.1%
**Systolic blood pressure**	117.4 (0.3)	118.3 (0.4)
**Hypercholesterolemia**	33.8%	38.7%
**LDL cholesterol**	115.8 (0.8)	122.9 (0.8)
**Work-related PA, min/day (self-reported)**	100.4 (4.3)	70.4 (3.5)

HCHS/SOL: Hispanic Community Health Study/Study of Latinos. PAG: 2018 Physical Activity Guidelines for Americans. BMI: body mass index. CESD-10: Center for Epidemiologic Studies Depression scale, 10 item. AHEI-2010: Alternative Healthy Eating Index 2010. LDL: low-density lipoprotein.

© 2024 Alver SK et al. *JAMA Network Open*.

eTable 2. Baseline characteristics by 2018 PAG Met/Not Met, FHS. FHS participant baseline characteristics by PAG Met/Not Met, FHS pooled sample (Gen 2/Gen 3/Omni 1/Omni 2/NOS). Continuous variables are reported as mean (SD) or median (Q1, Q3). Categorical variables are reported as n (%).

	2018 PAG Met (N=1062)	2018 PAG Not Met (N=2153)
**Female sex**	555 (52.3)	1248 (58.0)
**Age, yrs**	51.1 (12.4)	57.7 (13.9)
**BMI, kg/m^2**	26.0 (4.3)	28.0 (5.1)
**Employment Status [a], %**<br>**Retired**	129 (12.5)	436 (21.4)
**Not retired/not employed**	216 (10.6)	216 (10.6)
**Employed part-time**	165 (16)	327 (16)
**Employed full-time**	654 (63.5)	1061 (52)
**Annual Household Income [b], %**<br>**Under $20,000**	17 (1.6)	46 (2.1)
**$20,000-$34,999**	28 (2.6)	76 (3.5)
**$35,000-$54,999**	67 (6.3)	161 (7.5)
**$55,000-$74,999**	105 (9.9)	183 (8.5)
**$75,000-$100,000**	135 (12.7)	242 (11.2)
**Over $100,000**	371 (34.9)	395 (18.4)
**Prefer not to answer/Unknown**	339 (31.9)	1050 (48.8)
**Sleep duration, hours**	7.2 (0.9)	7.2 (1.1)
**Alcohol: Current (vs former/never)**	888 (88.1)	1600 (80)
**Smoking Status (Current vs Never/Former), %**	42 (4)	161 (7.5)
**Education Level [c], %**<br>**Less than high school**	3 (0.4)	14 (1.1)
**High school/GED**	48 (6.2)	173 (14.1)
**Some college, technical, associate**	158 (20.5)	394 (32.1)
**Bachelors, graduate, or professional**	561 (72.9)	645 (52.6)
**Years of education**	15.3 (2.5)	14.6 (2.6)
**Depression (CESD-20) [a]**	3.0 (1.0, 5.0)	3.0 (1.0, 6.0)
**AHEI-2010 [d]**	65.0 (13.1)	62.7 (12.8)
**Have health insurance (Yes vs No), %**	1026 (99.6)	2027 (99)
**Doctor visit in last year (Yes vs No), %**	967 (93.9)	1948 (95)
**SF-12 General Health score [a]**	3.2 (0.7)	2.9 (0.7)
**SF-12 Pain score [a]**	0.4 (0.7)	0.6 (0.8)
**Hypertension, %**	220 (20.7)	823 (38.2)
**Systolic blood pressure, mmHg**	117.0 (14.7)	121.2 (15.8)
**Hypercholesterolemia [e], %**	312 (29.4)	846 (39.3)
**LDL cholesterol, mg/dL**	103.7 (28.4)	106.1 (30.0)
**Moderate vigorous physical activity (MVPA), min/day**	29.5 (20.6, 44)	8.8 (3.0, 16.2)

[a] Employment status, SF-12 and CES-D not available for Omni 1 Exam 4
[b] Household income is reported for Gen 3/NOS/Omni 2
[c] Education level reported for Gen 3/NOS/Omni 1/Omni 2; years of education reported for Gen 2
[d]AHEI-2020 was not available for NOS/Omni 2
[e] Defined as total cholesterol ≥ 240 mg/dL or LDL ≥ 160 mg/dL or HDL < 40 mg/dL or use of lipid lowering drugs

FHS: Framingham Heart Study. PAG: 2018 Physical Activity Guidelines for Americans. BMI: body mass index. CESD-20: Center for Epidemiologic Studies Depression scale, 20 item. AHEI-2010: Alternative Healthy Eating Index 2010 score. LDL: low-density lipoprotein. MVPA: moderate to vigorous physical activity.

© 2024 Alver SK et al. *JAMA Network Open*.

eTable 3. Number of incident events stratified by prediabetes status, FHS and HCHS/SOL. Incident Events Stratified by Prediabetes Status, Framingham Heart Study (FHS) and Hispanic Community Health Study (HCHS/SOL) (unweighted counts/%).

**FHS Pooled Sample**	**Prediabetes (n=1028)**	**Normal (n=2739)**	**Overall (n=3767)**
	**N (col%)**	**N (col%)**	**N (%)**
All-cause mortality	40 (3.9)	59 (2.2)	99 (2.6)
CVD	66 (6.4)	81 (3)	147 (3.9)
MI	19 (1.9)	20 (0.7)	39 (1)
HF	7 (0.7)	22 (0.8)	29 (0.8)
Stroke/TIA	27 (2.6)	36 (1.3)	63 (1.7)

**HCHS/SOL**	**Prediabetes (n=4574)**	**Normal (n=4882)**	**Overall (n=9456)**
	**N (col%)**	**N (col%)**	**N (col%)**
All-cause mortality	100 (2.2%)	70 (1.5%)	170 (1.8%)
CVD	64 (1.4%)	27 (0.6%)	91 (1.0%)
MI	39 (0.9%)	9 (0.2%)	48 (0.5%)
HF	17 (0.4%)	5 (0.1%)	22 (0.2%)
Stroke/TIA	22 (0.5%)	16 (0.3%)	38 (0.4%)

Column % indicates cumulative incidence. CVD: cardiovascular disease. MI: Myocardial infarction. HF: Heart failure. TIA: transient ischemic attack.

© 2024 Alver SK et al. *JAMA Network Open*.

eTable 4. Estimated hazard ratio (95% confidence interval) for the association of the combined outcome of all-cause mortality or incident CVD event with binary PA/SB exposure by glycemic status, FHS and HCHS/SOL, minimally adjusted models. Among FHS and HCHS/SOL participants, estimated hazard ratio (95% confidence interval) for the association of the combined outcome of all-cause mortality or incident CVD event with binary PA/SB exposure by glycemic status. Results are from minimally adjusted models with binary PA exposure; n=9456 (HCHS/SOL, adjusted for age, sex, field center, Hispanic/Latino background), and n=3767 (FHS, adjusted for age and sex only), with prediabetes by PA or prediabetes by SB interaction term.

PA/SB measure	Prediabetes HR (95% CI)	Normoglycemia HR (95% CI)	Interaction p-value
**FHS**			
PAG not met vs met	1.08 (0.59, 1.98)	1.77 (1.05, 3.00)	0.22
**HCHS/SOL**			
PAG not met vs met	0.91 (0.58, 1.42)	1.33 (0.77, 2.28)	0.30
Steps <7000/d vs ≥ 7000/d	1.20 (0.77, 1.86)	1.32 (0.76, 2.28)	0.78
CPM low vs high	1.02 (0.67, 1.55)	1.21 (0.71, 2.06)	0.64
SB $2^{nd}/3^{rd}$ tertile vs 1st tertile	1.29 (0.78, 2.14)	2.02 (1.04, 3.92)	0.28

FHS: Framingham Heart Study. HCHS/SOL: Hispanic Community Health Study/Study of Latinos. CVD: cardiovascular disease. PA: Physical Activity. SB: sedentary behavior. PAG: 2018 Physical Activity Guidelines for Americans. CPM: Counts per minute.

eTable 5. Baseline characteristics at low and high sedentary behavior levels, HCHS/SOL.
HCHS/SOL participant baseline characteristics at low and high sedentary behavior (SB) levels, n=9456. Mean (SE) or percentage, adjusted for survey design.

	SB in Lowest Tertile (N=3124)	SB in 2nd/3rd Tertile (N=6332)
**Female sex**	50.2%	53.2%
**Age, yrs**	37.5 (0.4)	38.7 (0.3)
**BMI, kg/m^2**	28.5 (0.2)	29 (0.2)
**Employment Status**		
**Retired**	3.1%	5.8%
**Not retired/not employed**	36.8%	43.2%
**Employed part-time**	20.6%	17.1%
**Employed full-time**	39.5%	33.9%
**Annual Household Income**		
**Less than $10,000**	11.3%	12.5%
**$10,001-$20,000**	28.1%	28%
**$20,001-$40,000**	31%	30.8%
**$40,001-$75,000**	14.1%	13.5%
**More than $75,000**	6%	5.5%
**Not reported**	9.5%	9.7%
**Sleep Duration**	8.1 (0.04)	7.9 (0.03)
**Current Alcohol Use**	56%	53.9%
**Current Cigarette Use**	20.5%	21%
**Education level**		
**High school**	14.5%	15.9%
**<High school**	42%	43.4%
**Trade school**	14.8%	9.3%
**University/Other**	28.7%	31.4%
**Years of education**	12.3 (0.1)	12 (0.1)
**Language Preference (English)**	18.6%	30.7%
**Depression Symptoms (CESD-10)**	6.1 (0.1)	6.9 (0.1)
**AHEI-2010**	47.3 (0.3)	46.6 (0.2)
**Have health insurance**	38.1%	52.5%
**Doctor visit in last yr**	58.9%	65.8%
**Activity/work limited by health**	4%	6.6%
**SF-12 General Health Score**	2.7 (0.03)	2.8 (0.02)
**SF-12 Pain Score**	1.5 (0.02)	1.7 (0.03)
**Hypertension**	12.2%	15.7%
**Systolic blood pressure**	116.9 (0.4)	118.4 (0.3)
**Hypercholesterolemia**	36.8%	36.3%
**LDL cholesterol**	120.9 (0.8)	119 (0.7)
**Work-related PA, min/day (self-reported)**	107.7 (5.1)	71.9 (3.2)

HCHS/SOL: Hispanic Community Health Study/Study of Latinos. BMI: body mass index. CESD-10: Center for Epidemiologic Studies Depression scale, 10-item. AHEI-2010: Alternative Healthy Eating Index – 2010. LDL: low-density lipoprotein. PA: physical activity.

© 2024 Alver SK et al. *JAMA Network Open*.

eTable 6. Baseline characteristics at low and high counts per minute, HCHS/SOL. HCHS/SOL participant baseline characteristics at low and high counts per minute (CPM) levels (n=9456). Mean (SE) or percentage, adjusted for survey design.

	**CPM at/above median (N=4728)**	**CPM less than median (N=4728)**
**Female sex**	41.8%	63.5%
**Age, yrs**	36 (0.3)	40.8 (0.4)
**BMI, kg/m^2**	28.3 (0.1)	29.5 (0.2)
**Employment Status**		
**Retired**	2.9%	7%
**Not retired/not employed**	34.6%	48.1%
**Employed part-time**	20.7%	15.6%
**Employed full-time**	41.8%	29.3%
**Annual Household Income**		
**Less than $10,000**	10.3%	13.8%
**$10,001-$20,000**	28.3%	27.7%
**$20,001-$40,000**	32.5%	29.1%
**$40,001-$75,000**	15.1%	12.4%
**More than $75,000**	5.8%	5.6%
**Not reported**	8%	11.3%
**Sleep Duration**	7.9 (0.03)	8 (0.04)
**Current Alcohol Use**	58.8%	50.1%
**Current Cigarette Use**	21.2%	20.4%
**Education level**		
**High school**	14%	17%
**<High school**	44.9%	40.7%
**Trade school**	10.1%	12.3%
**University/Other**	30.9%	30%
**Years of education**	12.1 (0.1)	12 (0.1)
**Language Preference (English)**	28.8%	24.1%
**Depression Symptoms (CESD-10)**	6.3 (0.1)	7 (0.2)
**AHEI-2010**	47.2 (0.2)	46.5 (0.2)
**Have health insurance**	46.1%	49.1%
**Doctor visit in last yr**	61.7%	65.5%
**Activity/work limited by health**	4.2%	7.3%
**SF-12 General Health Score**	2.7 (0.02)	2.9 (0.03)
**SF-12 Pain Score**	1.5 (0.02)	1.7 (0.04)
**Hypertension**	11.6%	17.7%
**Systolic blood pressure**	117.2 (0.3)	118.6 (0.4)
**Hypercholesterolemia**	35.3%	37.8%
**LDL cholesterol**	117.2 (0.7)	122.4 (0.8)
**Work-related PA, min/day (self-reported)**	109.2 (4.2)	56.6 (3.3)

HCHS/SOL: Hispanic Community Health Study/Study of Latinos. BMI: body mass index. CESD-10: Center for Epidemiologic Studies Depression scale, 10-item. AHEI-2010: Alternative Healthy Eating Index – 2010. LDL: low-density lipoprotein. PA: physical activity.

© 2024 Alver SK et al. *JAMA Network Open*.

eTable 7. Baseline characteristics at steps ≥7000/d vs <7000/d, HCHS/SOL. HCHS/SOL participant baseline characteristics at steps ≥7000/d vs <7000/d (n=9421). Mean (SE) or percentage, adjusted for survey design.

	**Steps/day ≥7000 (N=4348)**	**Steps/day < 7000 (N=5073)**
**Female sex**	43.3%	59.5%
**Age, yrs**	38 (0.3)	38.5 (0.3)
**BMI, kg/m^2**	28.5 (0.2)	29.2 (0.2)
**Employment Status**<br>**Retired**	3.9%	5.6%
**Not retired/not employed**	34.3%	46.5%
**Employed part-time**	20.2%	16.7%
**Employed full-time**	41.6%	31.2%
**Annual Household Income**<br>**Less than $10,000**	12%	12%
**$10,001-$20,000**	28.8%	27.5%
**$20,001-$40,000**	30.9%	30.8%
**$40,001-$75,000**	13.9%	13.6%
**More than $75,000**	5.8%	5.6%
**Not reported**	8.6%	10.5%
**Sleep Duration**	7.8 (0.04)	8.1 (0.03)
**Current Alcohol Use**	56.2%	53.4%
**Current Cigarette Use**	20.1%	21.4%
**Education level**<br>**High school**	16.9%	14.3%
**<High school**	44.8%	41.4%
**Trade school**	9.7%	12.5%
**University/Other**	28.7%	31.9%
**Years of education**	11.8 (0.1)	12.3 (0.1)
**Language Preference (English)**	28%	25.3%
**Depression Symptoms (CESD-10)**	6.5 (0.1)	6.7 (0.1)
**AHEI-2010**	47.3 (0.2)	46.5 (0.2)
**Have health insurance**	49.6%	45.8%
**Doctor visit in last yr**	63.1%	63.8%
**Activity/work limited by health**	4.3%	6.9%
**SF-12 General Health Score**	2.8 (0.03)	2.8 (0.02)
**SF-12 Pain Score**	1.6 (0.04)	1.6 (0.02)
**Hypertension**	13.6%	15.3%
**Systolic blood pressure**	118.1 (0.4)	117.7 (0.3)
**Hypercholesterolemia**	36.4%	36.5%
**LDL cholesterol**	118.5 (0.8)	120.7 (0.8)
**Work-related PA, min/day (self-reported)**	106.9 (4.3)	65.4 (3.3)

HCHS/SOL: Hispanic Community Health Study/Study of Latinos. BMI: body mass index. CESD-10: Center for Epidemiologic Studies Depression scale, 10-item. AHEI-2010: Alternative Healthy Eating Index – 2010. LDL: low-density lipoprotein. PA: physical activity.

© 2024 Alver SK et al. *JAMA Network Open*.

eTable 8. (A) Estimated hazard ratios (95% confidence intervals) for the association of the combined outcome (all-cause mortality or incident CVD event) with continuous PA or SB exposures by glycemic status, HCHS/SOL; (B) Analogous HR for SB, steps and CAEE for combined glycemic groups. Among HCHS/SOL participants, estimated hazard ratios (95% confidence intervals) for the association of the combined outcome (all-cause mortality or incident CVD event) with continuous PA or SB exposures by glycemic status (n=9456).

eTable 8A

PA/SB measure	Prediabetes HR (95% CI)	Normoglycemia HR (95% CI)	Interaction p-value
MVPA per 30 min/day	1.12 (0.89, 1.41)	1.00 (0.78, 1.28)	0.46
CPM per 100 avg cpm/d	1.03 (0.86, 1.23)	0.99 (0.80, 1.21)	0.74
Steps per 500/day	0.99 (0.96, 1.02)	0.99 (0.95, 1.02)	0.87
SB per 30 min/d	1.05 (0.99, 1.12)	1.07 (0.98, 1.16)	0.64
CAEE per SD in kcal/d	0.88 (0.33, 2.35)	0.89 (0.39, 2.04)	--
CAEE per 50 kcal/d	0.98 (0.84, 1.15)	0.98 (0.86, 1.12)	--

Models for MVPA, CPM, steps and SB included a prediabetes by PA or prediabetes by SB interaction term and were adjusted for age, sex, field center, Hispanic/Latino background, SBP, LDL cholesterol, BMI, smoking status, alcohol use, education level, employment status, income, sleep duration, diet, language preference, wear time, recent doctor visit and use of health insurance. For CAEE, models were fit separately by glycemic status and adjusted for BMI, self-reported sedentary time and work, transportation or recreation-related PA, age, sex, LDL cholesterol, SBP, CESD-10, aggregate physical health score, employment status, income (including level for missing), 3-category Hispanic/Latino background, current smoking (vs former or never), high alcohol use (vs low or none), wear time, AHEI-2010, education level, sleep duration, language preference, doctor visit in the last year, and use of health insurance. CAEE models were fit separately by glycemic status, so there is no interaction p-value to report.

eTable 8B Among HCHS/SOL participants, estimated hazard ratios (95% confidence intervals) and p-values for the association of the combined outcome (all-cause mortality or incident CVD event) with continuous or binary PA or SB exposures for the combined glycemic groups (n=9456).

PA/SB measure	HR (95% CI)	p-value
Steps per 500/day	0.99 (0.96, 1.01)	0.32
Steps <7000/d vs ≥ 7000/d	1.24 (0.83, 1.86)	0.30
SB per 30 min/d	1.06 (0.99, 1.13)	0.10
SB 2nd and 3rd tertile vs 1st	1.33 (0.76, 2.35)	0.32
SB 2nd tertile vs 1st	1.32 (0.74, 2.37)	0.35
SB 3rd tertile vs 1st	1.84 (0.89, 3.80)	0.10
CAEE per SD in kcal/d	0.86 (0.23, 3.23)	0.69
CAEE per 50 kcal/d	0.98 (0.79, 1.21)	0.82

Models were adjusted for the same covariates listed for Table S.8a except for the interaction term. Prediabetes status was included as a covariate in the models.

HCHS/SOL: Hispanic Community Health Study/Study of Latinos. CVD: cardiovascular disease. PA: physical activity. SB: sedentary behavior. MVPA: moderate to vigorous physical activity. CPM: counts per minute. CAEE: calibrated activity-related energy expenditure. SBP: systolic blood pressure. LDL: low-density lipoprotein. BMI: body mass index. CESD-10: 10-item Center for Epidemiological Studies Depression Scale. AHEI: Alternative Healthy Eating Index.

© 2024 Alver SK et al. *JAMA Network Open*.

eTable 9. Estimated hazard ratios (95% confidence intervals), with additional exclusions as a sensitivity analysis, for the association of the composite outcome of all-cause mortality or first incident CVD event with PA levels (PAG not met as exposure vs met as reference) by glycemic status in HCHS/SOL. Among HCHS/SOL participants with additional exclusions as a sensitivity analysis, estimated hazard ratios (95% confidence intervals) for the association of the composite outcome of all-cause mortality or first incident CVD event with PA levels (PAG not met as exposure vs met as reference) by glycemic status.

**Result**	**No additional exclusions**	**Low BMI excluded**	**First year events excluded**	**First 2 years events excluded**
HR for PAG not met vs met, prediabetes	0.96 (0.59, 1.57)	0.93 (0.57, 1.51)	0.91 (0.55, 1.52)	0.88 (0.51, 1.50)
HR for PAG not met vs met, normoglycemia	1.37 (0.74, 2.55)	1.50 (0.79, 2.88)	1.31 (0.68, 2.52)	1.21 (0.61, 2.41)
Interaction p-value	0.39	0.24	0.40	0.47

Models were adjusted for age, sex, field center, Hispanic/Latino background, BMI, smoking, alcohol use, depression symptoms (CESD-10 score), sleep duration, AHEI-2010 score, years of education, work-related PA, SF-12 pain score, language preference, accelerometer wear time, SBP and LDL cholesterol. Models included a PA by prediabetes interaction term and accounted for complex survey design. The analysis included 214 events, 8446 participants after low BMI excluded; 205 events, 8506 participants after first year events excluded; 180 events, 8481 participants with first two years' events excluded. HCHS/SOL: Hispanic Community Health Study/Study of Latinos. CVD: cardiovascular disease. PA: physical activity. PAG: 2018 physical activity guidelines for Americans. BMI: body mass index. CESD-10: 10-item Center for Epidemiological Studies Depression Scale. AHEI: alternative healthy eating index - 2010. SF-12: 12-item short form health survey. SBP: systolic blood pressure. LDL: low-density-lipoprotein.

© 2024 Alver SK et al. *JAMA Network Open*.

eTable 10. Estimated hazard ratios (95% confidence intervals), with additional exclusions as a sensitivity analysis, for the association of the composite outcome of all-cause mortality or first incident CVD event with PA levels (PAG not met as exposure vs met as reference) by glycemic status in FHS. Among FHS participants with additional exclusions as a sensitivity analysis, estimated hazard ratios (95% confidence intervals) for the association of the composite outcome of all-cause mortality or first incident CVD event with PA levels (PAG not met as exposure vs met as reference) by glycemic status.

Result	No additional exclusions	Low BMI excluded	First year events excluded	First 2 years events excluded
PA HR, prediabetes	1.23 (0.61, 2.48)	1.17 (0.58, 2.37)	1.01 (0.49, 2.07)	1.02 (0.48, 2.17)
PA HR, normoglycemia	2.34 (1.25, 4.37)	2.22 (1.18, 4.16)	2.32 (1.24, 4.36)	2.40 (1.25, 4.62)
Interaction p-value	0.17	0.18	0.08	0.09

Models were adjusted for age, sex, BMI, smoking status, alcohol use, depression symptoms (CESD-10 score), sleep duration, AHEI-2010 score, SF-12 pain score, accelerometer wear time, SBP and LDL cholesterol. The analysis included 140 events, 2526 participants with low BMI excluded; 131 events, 3748 participants with first year events excluded; 120 events, 3731 participants with first two years' events excluded. FHS: Framingham Heart Study. CVD: cardiovascular disease. PA: physical activity. PAG: 2018 physical activity guidelines for Americans. BMI: body mass index. CESD-10: 10-item Center for Epidemiological Studies Depression Scale. AHEI: alternative healthy eating index - 2010. SF-12: 12-item short form health survey. SBP: systolic blood pressure. LDL: low-density-lipoprotein.

REFERENCES

1. Shaw, P.A., et al., *Calibration of activity-related energy expenditure in the Hispanic Community Health Study/Study of Latinos (HCHS/SOL).* J Sci Med Sport, 2019. **22**(3): p. 300-306.

2. Baldoni, P.L., et al., *On the Use of Regression Calibration in a Complex Sampling Design With Application to the Hispanic Community Health Study/Study of Latinos.* Am J Epidemiol, 2021. **190**(7): p. 1366-1376.

© 2024 Alver SK et al. *JAMA Network Open*.
