# Supplementary material for: Physical Activity, Cardiovascular Status, Mortality, and Prediabetes in Hispanic and Non-Hispanic Adults
Source: JAMA Netw Open. 2024 Jun 6;7(6):e2415094. doi: 10.1001/jamanetworkopen.2024.15094 (PMC11157354; doi:10.1001/jamanetworkopen.2024.15094)
Supplement: Supplement 2. — Data Sharing Statement [file jamanetwopen-e2415094-s002.pdf]

Data Sharing Statement

Alver. Physical Activity, Cardiovascular Status, Mortality, and Prediabetes in Hispanic and Non-Hispanic Adults. *JAMA Netw Open*. Published June 06, 2024.
doi:10.1001/jamanetworkopen.2024.15094

Data
Data available: No
